# Comparison of maternal-fetal attachment, anxiety, depression, and prevalence of intimate partner violence in Iranian women with intended and unintended pregnancy: a cross-sectional study

**DOI:** 10.1186/s40359-024-01847-x

**Published:** 2024-06-12

**Authors:** Mahsa Maghalian, Roghayeh Nikanfar, Mahsan Nabighadim, Mojgan Mirghafourvand

**Affiliations:** 1 Student Research Committee, Tabriz University of Medical Sciences, Tabriz, Iran; 2 Tabriz University of Medical Sciences, Tabriz, Iran; 3 Medical School, Ardabil University of Medical Sciences, Ardabil, Iran; 4 Social Determinants of Health Research Center, Tabriz University of Medical Sciences, Tabriz, Iran

**Keywords:** Pregnancy, Unplanned pregnancy, Domistic violence, Prevalence, Depression

## Abstract

**Background:**

Unintended pregnancies and intimate partner violence can adversely affect women, infants, and their psychological well-being. The study aimed to compare depression, anxiety, maternal-fetal attachment, and the prevalence of intimate partner violence between women with and without unintended pregnancies in Tabriz, Iran. The study sought to address the lack of research on this topic in the Iranian context.

**Methods:**

This cross-sectional study was conducted on 486 pregnant women attending health centers in Tabriz City between 2022 and 2023. A cluster sampling method was utilized, and data were gathered through the administration of socio-demographic, Maternal Fetal Attachment, Edinburgh Postnatal Depression, World Health Organization Domestic Violence, and Pregnancy Anxiety instruments. A general linear model (GLM), controlling for potential confounding variables, was used to compare anxiety, depression, and maternal-fetal attachment between the two groups. Multivariable logistic regression analysis, also controlling for potential confounding variables, was employed to compare the prevalence of domestic violence between the two groups.

**Results:**

The results of the adjusted GLM indicated that women with unintended pregnancies had significantly lower maternal-fetal attachment (Adjusted mean difference (AMD):-9.82, 95% CI:-12.4 to -7.15 ; *p* < 0.001)), higher levels of depression (AMD: 2.89; CI: 1.92 to 3.86 ; *p* < 0.001), and higher levels of anxiety (MD: 5.65; 95% CI: 3.84 to 7.45; *p* < 0.001) compared to women with intended pregnancies. During pregnancy, 40% of women with unintended pregnancies and 19.2% of women with intended pregnancies reported experiencing at least one form of physical, sexual, or emotional violence. The results of the adjusted multivariable logistic regression revealed that women with unintended pregnancies had a significantly higher odds of experiencing emotional violence (adjusted odds ratio [aOR]: 2.94; 95% CI: 1.64 to 5.26; *p* < 0.001), sexual violence, (aOR: 2.25; 95% CI: 1.32 to 3.85; *p* = 0.004), and physical violence (aOR: 2.38; 95% CI: 1.50 to 3.77; *p* < 0.001) compared to women with intended pregnancies.

**Conclusions:**

The study found that women with unintended pregnancies had lower levels of maternal-fetal attachment, higher levels of anxiety and depression, and a high prevalence of intimate partner violence, including physical, sexual, and emotional violence, compared to women with intended pregnancies. These results emphasize the importance of implementing policies aimed at reducing unintended pregnancies.

## Background

The global prevalence of unintended pregnancies between 2015 and 2019 was estimated to be around 48% [1, 2]. Unintended pregnancy has a significant impact on maternal mental health [3, 4]. A meta-analysis has demonstrated a significant statistical association between unintended pregnancy and the risk of postpartum depression [4]. The prevalence of depression during pregnancy is also reported to be twice as high in women with unintended pregnancies compared to those with intended pregnancies [5]. Depression during pregnancy can lead to lower birth weight, restricted fetal growth, preterm birth, preeclampsia, and an increased likelihood of cesarean delivery [6–8]. Additionally, women experiencing depression during pregnancy tend to have reduced maternal-fetal attachment [9].

Unintended pregnancy is associated with decreased maternal-fetal attachment [10] and can lead to inadequate prenatal care and nutrition, resulting in adverse outcomes for both the mother and the child [11]. The type of unintended pregnancy, whether it is mistimed or unwanted, has a significant relationship with maternal-fetal attachment, with mistimed pregnancies showing higher levels of attachment compared to unwanted pregnancies [12]. Maternal-fetal attachment can contribute to the pregnant woman’s adaptation to pregnancy. However, it has been shown that women who experience intimate partner violence have lower levels of maternal-fetal attachment, while greater support from their spouse leads to improvements in maternal-fetal attachment [13, 14]. Higher levels of maternal-fetal attachment are associated with better emotional, cognitive, and social development of the child [15].

Furthermore, women with unintended pregnancies experience higher levels of anxiety during pregnancy [16, 17]. Additionally, domestic violence itself leads to psychological distress for women [18]. Maternal anxiety during pregnancy is associated with negative outcomes for both the mother and the baby, including preterm birth, low birth weight, small-for-gestational-age infants, and reduced fetal head circumference [19].

Intimate partner violence is a global public health issue that refers to behavior by an intimate partner or former partner that causes physical, sexual, or psychological harm, including physical aggression, sexual coercion, psychological abuse, and controlling behaviors [20, 21]. Forced reproduction and sexual violence are significant contributors to unintended pregnancies. The risk of unintended pregnancy is higher among women who have experienced both physical and sexual abuse compared to those without such abuse. Additionally, the prevalence and severity of physical violence during pregnancy are higher among these women [2, 22]. However, there is limited research on the prevalence of intimate partner violence specifically among women with unintended pregnancies. Globally, more than one-third of women experience intimate partner violence [21], and in Iran, the prevalence of intimate partner violence among pregnant women has been reported to be 51.5% [23].

Among the risk factors for intimate partner violence are depression, poor mental and physical health, a partner’s substance abuse, living in a patriarchal family, having a partner who experienced childhood abuse, family tension, moderate family debt, and dysfunctional family dynamics [24]. During pregnancy, violence can have an impact on both the mother and the unborn child [25]. The health consequences associated with violence can manifest as physical harm, fetal miscarriage, and self-harming behaviors such as smoking. Additionally, it can contribute to conditions such as depression, anxiety, and substance abuse [26, 27].

The prevalence of intimate partner violence among pregnant women with unintended pregnancies has not been investigated in Iran. Existing studies have primarily focused on examining the prevalence of intimate partner violence in pregnant women, regardless of specific population groups, along with investigating related risk factors. However, some studies have indicated that unintended pregnancy is associated with a higher incidence of intimate partner violence [28–30]. Given the lack of comprehensive research on the prevalence of intimate partner violence among women with unintended pregnancies in Iran, the present study aimed to compare depression, anxiety, maternal-fetal attachment, and the prevalence of intimate partner violence among pregnant women with and without unintended pregnancies in Tabriz, Iran.

## Methods

### Study setting and participants

This cross-sectional study was conducted in Tabriz, Iran, from February 2022 to January 2023. Ethical approval was obtained from the Tabriz University of Medical Sciences (IR.TBZMED.REC.1400.681). The study included primigravida and multigravida women with healthy singleton pregnancies, aged between 20 and 40 week’s gestation. Exclusion criteria were: experiencing recent traumatic events, severe depression requiring medication before the recent pregnancy (as self-reported by participants and verified in records from the integrated healthcare system), and incomplete responses to more than 20% of the questionnaire items.

### Sample size calculation

The sample size was determined using the single proportion formula, based on the study by Naghizadeh et al. [31]. Considering a prevalence rate of 35.2% for domestic violence, a confidence level of 95%, and a study precision of 0.06, the initial sample size was estimated to be 243. Accounting for the cluster sampling design effect of 2, the final required sample size was calculated to be 486 individuals. The formula for sample size is as follows:

N=$$\frac{{Z}^{2}p\left(q\right)}{{d}^{2}}$$

### Data collection

The study used a cluster sampling method. Initially, one-fourth of the health centers in Tabriz city (21 health centers) were randomly selected using www.random.org. The researcher then visited these health centers and extracted a list of pregnant women between 20 and 40 weeks of gestation from the integrated healthcare system records. Based on the required sample size and the number of potential eligible women in each center, a number of these pregnant women were randomly chosen. The selected women were contacted by telephone, informed about the study objectives and methodology, and invited to attend their respective health center. During the face-to-face meeting, the study details were fully explained, and the questionnaires were completed through interviews conducted by the researcher.

### Measurements

For data collection in this study, the following instruments were used:

- Eligibility criteria checklist
- Socio-demographic characteristics questionnaire
- Maternal Fetal Attachment questionnaire
- Edinburgh Postnatal Depression Scale
- World Health Organization Domestic Violence Questionnaire
- Pregnancy Anxiety questionnaire

### Socio-demographic characteristics Questionnaire

This questionnaire included questions about the age, educational level, employment status of the women and their spouses, and the sufficiency of household income. The qualitative content validity of this questionnaire was established through assessment by faculty members of the Nursing and Midwifery college of Tabriz University of Medical Sciences.

### Maternal-infant attachment scale

This scale comprises 24 questions, which are rated on a scale of 1 to 5. Notably, item 22 is scored in reverse. The obtained scores range between 24 and 120, with higher scores indicating a higher level of maternal-fetal attachment [32]. The Persian version of the scale has been evaluated for validity and reliability, yielding a Cronbach’s alpha coefficient of 0.80, indicating good internal consistency [33].

### Edinburgh postnatal depression scale (EPDS)

This questionnaire comprises 10 questions, rated on a 4-point Likert scale ranging from 1 to 4. The total EPDS score ranges from 0 to 30. Questions 1, 2, and 4 are scored from 0 to 3, while questions 3, 5, 6, 7, 8, 9, and 10 are scored from 3 to 0 [34]. The validity of the EPDS questionnaire was established in Iran by Ahmadi-Kani Golzar et al. (2015), who reported a Cronbach’s alpha coefficient of 0.70, indicating good internal consistency [35].

### Pregnancy anxiety questionnaire

This questionnaire, designed by Huizink et al., assesses anxiety levels during pregnancy. It comprises 11 items that are categorized into three factors: fear of childbirth (3 items), concerns about having a child with physical or mental disabilities (4 items), and worries about physical changes (3 items). Each item is rated on a 5-point Likert scale [36]. The Persian version of the questionnaire was evaluated for validity and reliability by Bayrampour et al., who reported a reliability coefficient of 0.74 for the entire instrument, indicating acceptable reliability [37].

### The intimate partner violence questionnaire

The intimate partner violence questionnaire was designed by the World Health Organization. It measures the violence applied by the partner in three areas: physical, sexual, and emotional. The questionnaire includes 10 questions on physical violence, 5 questions on sexual violence, and 11 questions on emotional violence. The number of cases of violence was calculated based on a 5-point Likert scale. A woman was considered to have experienced violence if she gave at least one positive answer to any of the questions related to physical, sexual, or emotional violence [38]. In a previous study by Naghizadeh et al., the Cronbach’s alpha coefficient was 0.70, 0.78, and 0.79 respectively for the three domains of physical, emotional, and sexual violence of the questionnaire, indicating good internal consistency [31].

### Statistical analysis

After collecting data from the participants, the data were analyzed using SPSS-Version24 software. Independent t-tests, Chi-square test for trend, and Fisher’s exact tests were used to compare socio-demographic characteristics between the two groups: women with unintended pregnancies and women with intended pregnancies.

The key assumptions of the General Linear Model (GLM) include linearity, homoscedasticity (constant variance), normality, and independence. The normality of the data was evaluated using measures of skewness and kurtosis, and the results indicated that all data variables had a normal distribution. One of the crucial assumptions for the appropriateness of the GLM is the normality of the residuals [39]. The normality of the residuals was examined by constructing a Normal Probability Plot, which suggested that the use of the GLM was appropriate.

In the GLM model, unintended pregnancy was entered as the independent variable, while maternal-fetal attachment, anxiety, and depression were included as the dependent variables. Furthermore, total violence and various types of violence during pregnancy were entered as dependent variables, and unintended pregnancy was also incorporated as an independent variable in the multiple logistic regression analysis.

Socio-demographic characteristics with a p-value less than 0.2 that were associated with the dependent variables were included in the GLM and multiple logistic regression models as potential confounding factors.

## Results

A total of 563 women were screened for eligibility to participate in the study. Of these, 54 did not meet the inclusion criteria, and 19 declined to participate. Ultimately, 486 women were enrolled and included in the present study.

Among the socio-demographic characteristics, only the age of the women and their spouses showed a significant difference between the two groups. Women with unintended pregnancies had a higher mean age (29.57 years, standard deviation (SD) = 6.67) compared to women with intended pregnancies (27.59 years, SD = 6.66). Similarly, the spouses of women with unintended pregnancies had a higher mean age (34.64 years, SD = 6.44) compared to the spouses of women with intended pregnancies (33.23 years, SD = 6.07). Other characteristics, such as job, educational level of women and their spouses, and family income sufficiency, did not differ significantly between the two groups of unintended and intended pregnancies (Table 1).

**Table 1:** Comparison of participants’ socio-demographic characteristics based on unintended and intended pregnancies

Continuse variables	Unintended pregnancy (*n* = 150)		Intended pregnancy (*n* = 336)	*P* value
	Mean (SD)		Mean (SD)	
**Age** (Years)	29.57 (6.67)		27.59 (6.66)	**0.003** ^**a**^
**Spouse’s age** (Years)	34.64 (6.44)		33.23 (6.07)	**0.025** ^**a**^
**Categorical variables**	**Number (percent)**		**Number (percent)**	
**Job**				0.666 ^b^
Housewife	132 (88.0)		292 (86.4)	
Employed	18 (12.0)		46 (13.6)	
**Educational level**				0.914 ^c^
Elementary	38 (25.3)		109 (32.2)	
Secondary	46 (30.7)		76 (22.5)	
High school	29 (19.3)		66 (19.5)	
Diploma	22 (14.7)		44 (13.0)	
University	15 (10.0)		43 (12.7)	
**Spouse’s job**				0.313 ^b^
Worker	39 (26.0)		97 (28.7)	
Employee	20 (13.3)		63 (18.6)	
Shopkeeper	7 (4.70)		18 (5.30)	
Self-employment	84 (56.0)		160 (47.3)	
**Spouse’s education level**				0.415 ^c^
Elementary	13 (8.70)		37 (10.9)	
Secondary	21 (14.0)		55 (16.3)	
High school	19 (12.7)		48 (14.2)	
Diploma	57 (38.0)		100 (29.6)	
University	40 (26.7)		98 (29.0)	
**Sufficiency of family income**				0.402 ^c^
Completely sufficient	13 (8.70)		18 (5.30)	
Relatively sufficient	113 (75.3)		263 (77.8)	
Insufficient	24 (16.0)		57 (16.9)	

a = Independent t-tests, b = Fisher’s exact tests, c = Chi-square tests for trend

In women with unintended pregnancies, the mean (SD) scores for maternal-fetal attachment, anxiety, and depression were 79.4 (17.7), 30.4 (9.58), and 10.6 (5.30), respectively. In women with intended pregnancies, these scores were 89.3 (11.7), 24.8 (8.77), and 7.5 (5.08), respectively.

The results of the independent t-test for maternal-fetal attachment showed that women with unintended pregnancies had significantly lower maternal-fetal attachment scores compared to women with intended pregnancies (mean difference [MD]: -9.87; 95% confidence interval [CI]: -12.5 to -7.20 ; *p* < 0.001). Additionally, the results of the adjusted GLM, which was adjusted for the woman’s job, also showed similar findings (Adjusted mean difference (AMD):-9.82, 95% CI:-12.4 to -7.15 ; *p* < 0.001).

The results of the independent t-test for anxiety showed that women with unintended pregnancies had significantly higher anxiety scores compared to women with intended pregnancies (MD: 5.65; 95% CI: 3.84 to 7.45; *p* < 0.001). There was no significant relationship between anxiety and sociodemographic characteristics. As a result, adjusted analysis was not conducted.

The results of the independent t-test for depression showed that women with unintended pregnancies had significantly higher depression scores compared to women with intended pregnancies (MD: 3.18; 95% CI: 2.18 to 4.18; *p* < 0.001). Additionally, the results of the adjusted GLM, which was adjusted for the woman’s age, her spouse’s age, the woman’s employment status, the woman’s educational level, and the spouse’s employment status, also showed similar findings (AMD: 2.89; CI: 1.92 to 3.86 ; *p* < 0.001) (Table 2).

**Table 2:** Maternal-fetal attachment, anxiety, depression, and violence during pregnancy among women with unintended pregnancies compared to women with intended pregnancies

Variable	Unintended pregnancy (*n* = 150)	Intended pregnancy (*n* = 336)	Unadjusted anlysis		Adjusted anlysis	
Continuse variables	Mean (SD)	Mean (SD)	MD (95%CI)	*P* value	AMD (95%CI)	*P* value
**Maternal-Fetal Attachment**	79.4 (17.7)	89.3 (11.7)	-9.87 (-12.5 to -7.20)	< 0.001 ^**a**^	-9.82 (-12.4 to -7.15)	< 0.001 ^b^
**Anxiety**	30.4 (9.58)	24.8 (8.77)	5.65 (3.84 to 7.45)	< 0.001 ^**a**^	5.65 (3.84 to 7.45)	< 0.001
**Depression**	10.6 (5.30)	7.5 (5.08)	3.18 (2.18 to 4.18)	< 0.001 ^**a**^	2.89 (1.92 to 3.86)	< 0.001 ^c^
**Categorical variables**	**n (%)**	**n (%)**	**OR (95%CI)**	**P value**	**aOR (95%CI)**	**P value**
**Total violence**	60 (40.0)	65 (19.2)	2.80 (1.83 to 4.27)	< 0.001	2.76 (1.80 to 4.25)	< 0.001 ^d^
**Physical violence**	47 (31.3)	53 (15.7)	2.45 (1.56 to 3.87)	< 0.001	2.38 (1.50 to 3.77)	< 0.001 ^d^
**Sexual violence**	31 (20.7)	34 (10.1)	2.32 (1.37 to 3.96)	0.002	2.25 (1.32 to 3.85)	0.004 ^e^
**Emotional violence**	28 (18.7)	25 (7.40)	2.87 (1.61 to 5.12)	< 0.001	2.94(1.64 to 5.26)	< 0.001 ^f^

a = Independent t test; b = Adjusted for woman’s Job; c = Adjusted for age of the woman and her spouse, woman’s job, woman’s educational level, spouse’s job; d = Adjusted for age of the woman and her spouse; e = Adjusted for spouse’s age; f = Adjusted for sufficiency of family income;

CI = Confidence interval; SD = Standard deviation; MD = mean difference; OR = odds ratio

Score range: 24–120 for maternal-fetal attachment, 11–55 for anxiety, and 0–30 for depression

During pregnancy, 40% of women with unintended pregnancies and 19.2% of women with intended pregnancies reported experiencing at least one form of physical, sexual, or emotional violence. Among these groups, the rates of physical violence were 31.3% for unintended pregnancies and 15.7% for intended pregnancies. This was followed by sexual violence, which occurred at rates of 20.7% for unintended pregnancies and 10.1% for intended pregnancies. Additionally, emotional violence was reported by 18.7% of women with unintended pregnancies and 7.4% of women with intended pregnancies.

The results of the adjusted multivariable logistic regression analysis showed that women with unintended pregnancies had significantly higher odds of experiencing total violence, adjusted for the woman’s age and her spouse’s age (adjusted odds ratio [aOR]: 2.76; 95% CI: 1.80 to 4.25; *p* < 0.001), physical violence, adjusted for the woman’s age and her spouse’s age, (aOR: 2.38; 95% CI: 1.50 to 3.77; *p* < 0.001), sexual violence, adjusted for the spouse’s age (aOR: 2.25; 95% CI: 1.32 to 3.85; *p* = 0.004), emotional violence, adjusted for the sufficiency of family income (aOR: 2.94; 95% CI: 1.64 to 5.26; *p* < 0.001) compared to women with intended pregnancies. The unadjusted multivariable logistic regression analysis also showed similar results for women with unintended pregnancies, with significant associations for total violence (*p* < 0.001), physical violence (*p* < 0.001), sexual violence (*p* = 0.002), and emotional violence (*p* < 0.001) (Table 2).

## Discussion

The results of this study showed that women with unintended pregnancies had significantly lower maternal-fetal attachment and higher levels of anxiety and depression compared to women with intended pregnancies.

These findings are consistent with the existing evidence. A systematic review and meta-analysis of 15 studies involving 41,054 participants demonstrated that unintended pregnancy increased the likelihood of depression during pregnancy by 1.59 times (95% CI: 1.35 to 1.92) compared to intended pregnancy [40]. Several other recent studies, which were not included in this meta-analysis, have also found a significant association between unintended pregnancy and both depression and anxiety. For example, a cohort study involving 1,928 women in the Netherlands showed that women with unintended pregnancies had higher levels of depressive symptoms in each trimester of pregnancy and up to 12 months postpartum, compared to women with intended pregnancies [41]. Similarly, another Dutch study indicated that carrying an unintended pregnancy to term, and the associated distress and upset, could lead to symptoms of depression, anxiety, and stress during pregnancy and even up to 5 years after childbirth [10].

Studies have shown that women with higher levels of depression, anxiety, and stress tend to experience a weaker maternal-fetal bond during pregnancy [42, 43]. The parent-infant bond that develops during the prenatal period involves emotional, behavioral, cognitive, and neurobiological connections between parents and the fetus [44]. Research has found a positive correlation between lower acceptance of pregnancy and higher levels of depression/anxiety, as well as a weaker maternal-fetal bond during pregnancy [45]. Furthermore, women with unintended pregnancies have been found to have a weaker mother-fetal bond during pregnancy, and this is correlated with a weaker mother-infant bond after birth, suggesting the prenatal bond is a protective factor [46]. This additional context helps further elucidate the mechanisms behind the lower maternal-fetal attachment observed in women with unintended pregnancies. It highlights how psychological factors like depression, anxiety, and stress can negatively impact the development of the critical prenatal parent-child bond.

The present study found that two out of every five women with unintended pregnancies had experienced intimate partner violence (physical, sexual, and emotional) by their partners. This prevalence is similar to the 36% reported in a previous study by Tiruye et al. [47]. Additionally, a significant association was found between intimate partner violence and unintended pregnancy.

Among women with both intended and unintended pregnancies in the current study, the highest odds of violence was emotional violence, while the lowest was sexual violence. This finding is consistent with a systematic review and meta-analysis of Iranian pregnant women, which reported 43.2% for emotional violence (the highest type) [23]. Similarly, a recent United States study found a low overall prevalence of intimate partner violence during pregnancy (5.7%), but emotional violence was the most commonly reported type [48]. This underscores the complex and multifaceted relationship between unintended pregnancy, intimate partner violence, and maternal mental health.

Violence was also more prevalent among women with unintended pregnancies and those experiencing depression, suggesting a significant association between domestic violence and depression during pregnancy [49, 50]. The disparities in domestic violence prevalence between Iran and the United States may be attributed to a few key factors, including differences in the measurement tools used to assess domestic violence, variations in sample sizes across studies, as well as the reluctance of women to disclose their experiences of abuse.

Domestic violence during pregnancy can have significant adverse impacts on both the woman and her newborn child [51]. Studies have shown it can lead to psychological issues for the woman, as well as disruptions in birth outcomes like low birth weight and preterm delivery [52, 53]. Notably, emotional form of domestic violence during pregnancy appear to increase the woman’s risk of developing conditions like post-traumatic stress disorder and depression [54]. This is likely due to factors such as reduced social support and lower spousal engagement during this vulnerable time [55]. Interestingly, unplanned pregnancy itself can also contribute to increased depression in women. Furthermore, domestic violence during pregnancy has been linked to reduced maternal-fetal bonding and attachment [55, 56]. Overall, the complex interplay of unplanned pregnancy, lack of social support, and psychological domestic violence appears to be a key driver of these negative outcomes.

Interventional studies with appropriate methodologies should also be implemented to examine ways to improve anxiety, depression, and maternal-fetal attachment in women with unintended pregnancies. Such interventions may include educational programs on effective pregnancy prevention methods, counseling services, or increasing awareness among partners through educational initiatives to promote collaboration and support during unintended pregnancies. By implementing evidence-based policies and interventions, healthcare providers and policymakers can work to improve outcomes for women experiencing unintended pregnancies and exposure to domestic violence.

### Strengths and limitations

Investigating the prevalence of intimate partner violence among women with unintended pregnancies for the first time in Iran, as well as examining several outcomes related to unintended pregnancy in a comprehensive study using standardized tools, are among the strengths of the current study. Additionally, the random selection of women to participate in the study adds to its strengths.

One of the limitations of the present study is its exclusive focus on women of Azeri ethnicity, which restricts the generalizability of the findings to other ethnic groups. Therfore, further studies should be conducted among other ethnic groups to improve the generalizability of the findings. Additionally, the cross-sectional nature of the study design precludes establishing a definitive cause-effect relationship between maternal-fetal attachment, anxiety, depression, and intimate partner violence in the context of unintended pregnancies.Given the limitations in following up with women in the present study to assess birth and neonatal outcomes, it is recommended that future studies examine the neonatal and delivery outcomes for women with unintended pregnancies who also experience domestic violence.

## Conclusions

The study found that women with unintended pregnancies had lower levels of maternal-fetal attachment, higher levels of anxiety and depression, and a high prevalence of intimate partner violence, including physical, sexual, and emotional violence, compared to women with intended pregnancies. Therefore, considering the impact of unintended pregnancy on women’s emotional and psychological well-being, as well as the high prevalence of domestic violence experienced by these women, it is crucial to implement policies aimed at reducing unintended pregnancies. The complex interplay between unintended pregnancy, intimate partner violence, and maternal mental health underscores the need for a multifaceted approach to address this critical public health issue.

## Data Availability

The corresponding author can provide the datasets used and analyzed in the current study upon a reasonable request.

## Abbreviations

OR: Odds Ratio
CI: Confidence Interval
EPDS: Edinburgh Postnatal Depression Scale
GLM: General Linear Model
MD: Mean Difference
SD: Standard Deviation
